# Effect of Diamorphine on Spatial Learning and Memory and Mitochondrial Function of Male Rat Brain

**DOI:** 10.5812/ijpr-162320

**Published:** 2025-07-16

**Authors:** Ainaz Moshtagh, Maryam Mehdizadeh, Ghodsieh Hosseinifakhr, Alireza Foroumadi, Maryam Baeeri, Shokoufeh Hassani, Mahdi Gholami, Zahra Emamgholipour, Omid Sabzevari, Rohollah Hosseini, Abbas Kebriaeezadeh, Ghorban Taghizadeh, Mohammad Sharifzadeh

**Affiliations:** 1 Department of Toxicology and Pharmacology, Faculty of Pharmacy, Tehran University of Medical Sciences, Tehran, Iran; 2 Geriatric Mental Health Research Center, Iran University of Medical Sciences (IUMS), Tehran, Iran; 3 Department of Medicinal Chemistry, Faculty of Pharmacy, Tehran University of Medical Sciences, Tehran, Iran; 4 Toxicology and Diseases Group (TDG), Pharmaceutical Sciences Research Center (PSRC), The Institute of Pharmaceutical Sciences (TIPS), Tehran University of Medical Sciences (TUMS), Tehran, Iran; 5 Department of Toxicology and Pharmacology, Toxicology and Poisoning Research Centre, Faculty of Pharmacy, Tehran University of Medical Sciences, Tehran, Iran; 6 Department of Occupational Therapy, Rehabilitation Research Center, School of Rehabilitation Sciences, Iran University of Medical Sciences, Tehran, Iran; 7 Department of Toxicology and Pharmacology, Faculty of Pharmacy, and The Institute of Pharmaceutical Sciences (TIPS), Tehran University of Medical Sciences, Tehran, Iran

**Keywords:** Diamorphine, Mitochondrial Impairment, Spatial Learning and Memory, Oxidative Stress, Reactive Oxygen Species, Lipid Peroxidation

## Abstract

**Background:**

Opioid abuse is a global crisis, with diamorphine being one of the most dangerous substances of abuse. Diamorphine is a major contributor to addiction and social issues.

**Objectives:**

This study aimed to investigate the impact of different doses of diamorphine on spatial learning and memory by examining its effects on brain mitochondria function.

**Methods:**

Four groups of nine rats were selected to receive diamorphine at doses of 1, 5, and 10 mg/kg, while one group received diamorphine solvent at a dose of 1 mL/kg. All treatments were given twice a day at 12-hour intervals for 10 days. The animals' memory performance was assessed using the Morris Water Maze test. Additionally, tests were conducted to measure ADP/ATP levels, mitochondrial reactive oxygen species (ROS) levels, mitochondrial membrane potential (MMP), lipid peroxidation (LPO), and antioxidant function, including total thiol groups measurement (TTM), and ferric reducing antioxidant power (FRAP).

**Results:**

The results indicated that diamorphine at doses of 5 and 10 mg/kg significantly disrupted learning and spatial memory, as evidenced by changes in latency (P < 0.0001), distance (P < 0.0001), and time spent in the target quadrant (P < 0.0001). Diamorphine also negatively impacted mitochondrial function parameters, such as ROS levels (P < 0.0001), MMP (P < 0.0001), mitochondrial swelling (P < 0.0001), and ADP/ATP ratio (P < 0.0001). Furthermore, brain antioxidant capacity was compromised (P < 0.0001).

**Conclusions:**

This study on the mechanisms of brain damage induced by diamorphine showed that the harm arises from the impairment of mitochondrial function. This impairment leads to the generation of ROS, reduced antioxidant capacity, decreased MMP, and an elevated ADP/ATP ratio.

## 1. Background

Opioid use disorder is a pressing global concern. As individuals develop tolerance and dependence on opioids, they experience a myriad of problems and crises throughout their lives (1). Among opioids, diamorphine (heroin) has emerged as one of the main causes of addiction, social problems, and costs. People who suffer from diamorphine abuse and dependence constitute an important population of substance abuse patients (2). Diamorphine is the most widely used opioid in the world and, compared to morphine, it acts more potently in creating euphoria and relieving pain (3). About 2.1 million Americans are suffering from opioid use disorder, with approximately 1 million of them abusing diamorphine, and 4.8 million people have used diamorphine at some point in their lives (4). In a national study in Iran in 1993, the total number of drug users was 7500 per 100,000 population. Numerous studies from 1991 - 1998 have shown that drug addiction is one of the most important problems in society, with a prevalence of 16.5% in Iran (5). Diamorphine (also known as 3 and 6 diacetylmorphine) is a semi-synthetic drug obtained by acetylating two hydroxyl groups in morphine (6). According to a study, diamorphine plays an important role in deaths caused by opioid use, accounting for about 32% of deaths caused by drug abuse referred to the Legal Medicine Organization (LMO) in Iran in 2015 (7). Diamorphine is a common substance of abuse that causes deficits in attention and weaker performance in memory tasks (5). Chronic use of opioids such as diamorphine and morphine has been shown to impair cognitive function (8, 9). Studies have also shown that compared to morphine, diamorphine has the potential for more neurotoxic effects, increasing the activity of caspase 3 and inducing DNA damage, protein, and lipid oxidation (10). By inhibiting mitochondrial complex 1 through the activation of mitochondrial permeable pores in neurons, morphine causes excessive production of reactive oxygen species (ROS). This oxidative damage can affect different parts of the brain, including the cerebral cortex and hippocampus, which are involved in memory and learning (11).

## 2. Objectives

The aim of the present study is to examine the effects of different doses of diamorphine on spatial memory and learning through its effects on brain mitochondria function.

## 3. Methods

### 3.1. Animals

 Adult male Wistar rats (200 - 250 g) were provided by the Faculty of Pharmacy of Tehran University of Medical Sciences (TUMS). We kept the rats in cages during the experiment with a 12-hour day/night cycle, with free access to food and water. Body weight was monitored throughout the experiment. The animals were randomly divided into three groups of 9 rats each: Group A for mitochondrial tests [ROS, mitochondrial membrane potential (MMP), Swelling], group B for assessing the antioxidant state of the rat brain [total thiol groups measurement (TTM), ferric reducing antioxidant power (FRAP), lipid peroxidation (LPO)], and group C for the ADP/ATP ratio test.

### 3.2. Materials

Diamorphine was synthesized from morphine sulfate using the method described by Barton et al. (12), and the resulting diamorphine structure was confirmed using nuclear magnetic resonance (NMR) spectroscopy. The chemicals used included 4-(2-Hydroxyethyl)-1-piperazine ethanesulfonic acid (HEPES), Trizma–HCl, sodium succinate, 2',7'-dichlorodihydrofluorescein diacetate (DCFH-DA), thiobarbituric acid (TBA), dimethyl sulfoxide (DMSO), rotenone, Coomassie Brilliant Blue G 250, ferrous chloride (FeCl_2_), ethylene glycol-bis[beta-aminoethyl ether (EGTA)-N,N,N',N'-tetraacetic acid], potassium chloride, 3-(4,5-dimethylthiazol-2-yl)-2,5-diphenyl tetrazolium bromide (MTT), sucrose, MgCl_2_, rhodamine123 (Rh123), Na_2_HPO_4_, 2,4,6-tri(2-pyridyl)-s-triazine (TPTZ), dithiobis nitro benzoic acid (DTNB), xylazine (Royan Darou, Tehran, Iran), and ketamine (Rotexmedica, Bunsenstr. 4. 22946 Trittau, Germany).

### 3.3. Experiments

In order to create a model of addiction and memory destruction caused by opioids, male rats were subcutaneously injected with diamorphine twice a day with a 12-hour interval for ten consecutive days. All animal studies were conducted in accordance with international and national guidelines, including the U.K. Animals (Scientific Procedures) Act, 1986, and EU Directive 2010/63/EU for animal experiments. Additionally, experiments were reported according to the Animal Research: Reporting of In Vivo Experiments (ARRIVE) guidelines. All procedures were carried out under the supervision of the Ethics Committee of TUMS with approval code IR.TUMS.VCR.REC.1398.049.

For this study, 36 male Wistar rats weighing between 200 - 250 grams, with free access to water and standard food, were included. The animals were randomly assigned to one of four groups, each consisting of 9 male rats. The diamorphine dose and injection timing were based on previous studies (13, 14). Diamorphine was dissolved in normal saline in three doses of 1, 5, and 10 mg/kg (injection volume up to 1 mL/kg) and immediately injected subcutaneously twice a day at 12-hour intervals.

The groups received the following treatments:

1. Control group: Received diamorphine solvent (normal saline) at a dose of 1 mL/kg via subcutaneous injection for 10 days (twice a day, 12-hour intervals).

2. Group receiving diamorphine at a dose of 1 mg/kg: Administered via subcutaneous injection for 10 days (twice a day, 12-hour intervals).

3. Group receiving diamorphine at a dose of 5 mg/kg: Administered via subcutaneous injection for 10 days (twice a day, 12-hour intervals).

4. Group receiving diamorphine at a dose of 10 mg/kg: Administered via subcutaneous injection for 10 days (twice a day, 12-hour intervals).

Six hours after the last injection, the memory status of the animals was evaluated using the Morris Water Maze. The maze used in this study had a pond with a diameter of 180 cm and a depth of 70 cm, filled halfway with water at a temperature of 22 - 26 degrees Celsius. The pond was hypothetically divided into four quadrants: North-east (NE), north-west (NW), south-east (SE), and south-west (SW). Four starting points were designated as north (N), east (E), south (S), and west (W). Ten millimeters below the water level, in the center of the southeast quadrant, a transparent plexiglass platform with a diameter of one hundred millimeters was placed. The platform remained hidden and in the same position throughout the training days. Visual cues were present in the room for the animals during the experiment.

The training phase consisted of four trials on the first day. If an animal found the platform within 90 seconds, it would stay on it for 30 seconds before the next trial. Animals that did not find the platform within 90 seconds were gently guided to it and placed on it for 30 seconds. Training was conducted for four days with four trials each day at the same time. On the fifth day, a single trial (probe test) was conducted without the platform. The animal was allowed to swim in the pool for 90 seconds, and the time spent in the quadrant where the platform used to be was recorded (15, 16). The animal's movements in the maze were recorded by a camera placed above the maze and analyzed using Ethovision software, version 11.5 (Noldus, Wageningen, Gelderland, The Netherlands).

#### 3.3.1. Mitochondria Isolation Technique from Brain

Following the completion of the probe test, nine rats were promptly selected from each experimental group and equally divided into three subgroups (n = 3 per subgroup). One subgroup was designated for the ADP/ATP assay, another was utilized to assess mitochondrial functional parameters, and the third subgroup was assigned to LPO and antioxidant function tests, including TTM and FRAP. The rats were anesthetized with ketamine (100 mg/kg) and xylazine (10 mg/kg), then sacrificed to collect brain tissues. Brain mitochondria were isolated using the differential centrifugation technique after homogenizing the whole-brain tissue with a manual glass homogenizer (17). During the first centrifugation step (at 1500 g for 10 min, 4°C), cell debris and nuclei were removed. The supernatant obtained was then subjected to a second centrifugation step at 10,000 g for 10 min, 4°C. The resulting supernatant was discarded, and isolation medium was added to the mitochondrial pellets. This was followed by another 10,000 g centrifugation step for 10 min, at 4°C. The isolated mitochondria pellets were then suspended in Tris buffer (0.25 M sucrose, 0.05 M Trizma HCl, 2.0 mM magnesium chloride, 20 mM potassium chloride, and 1.0 mM disodium hydrogen phosphate, pH 7.4) at 4°C.

For the measurement of ROS levels, the mitochondria samples were suspended in a respiration buffer containing 320 μM sucrose, 10 mM Tris, 20 mM Mops, 50 μM EGTA, 0.5 mM MgCl_2_, 0.1 mM KH2PO4, and 5 mM sodium succinate. For MMP, the samples were suspended in a buffer containing 220 mM sucrose, 5 mM monobasic potassium phosphate, 10 mM potassium chloride, 68 mM D-mannitol, 0.002 M magnesium chloride, 10 mM HEPES, 0.005 M succinate, 0.05 mM EGTA, and 2 μM Rotenone. The samples isolated for the assessment of mitochondrial swelling were suspended in a swelling buffer containing 0.07 M sucrose, 3 mM HEPES, 230 mM mannitol, 0.002 M Tris-phosphate, 5 mM succinate, and 0.001 mM rotenone. The Bradford method (Bradford, 1976) was then used to measure protein concentration and determine the amount of brain mitochondrial protein. Samples with a protein concentration of 500 μg/mL were used in all experiments (18).

#### 3.3.2. Measurement of Brain Mitochondrial Reactive Oxygen Species Level

This test is based on the reaction between oxidizing agents in the mitochondria and DCFH-DA, which is measured using a fluorescence spectrophotometer. Incubation and quantification were carried out following the methods previously published (17-20), using a Synergy 4 multi-mode microplate reader (Biotek, Winooski, Vermont, USA) with an excitation wavelength of 498 nm and an emission wavelength of 522 nm.

#### 3.3.3. Measurement of Brain Mitochondrial Membrane Potential

Brain MMP was measured by the mitochondrial uptake of Rh123. Briefly, Rh123 was added to brain mitochondrial fractions at a concentration of 10 μM in the MMP assay buffer described previously. The fluorescence was then measured using a fluorescence spectrophotometer (Shimadzu RF5000U, Shimadzu, Kyoto, Japan), with excitation and emission wavelengths of 490 nm and 535 nm, respectively (21).

#### 3.3.4. Determination of Brain ADP/ATP Ratio

To measure the ADP/ATP ratio, high-performance liquid chromatography (HPLC) was performed. Brain tissue was homogenized in a 1 mL 6% TCA solution, then centrifuged for 10 minutes at 12,000 g at 4°C. After neutralizing the supernatant with potassium hydroxide, the HPLC experiment was conducted as described previously (22).

#### 3.3.5. Measurement of Brain Mitochondrial Swelling

This test was conducted using a spectrophotometer set to a wavelength of 540 nm, and the temperature of the suspension containing mitochondria was 30°C. A decrease in light absorption indicated an increase in the swelling of the mitochondria (17).

#### 3.3.6. Brain Antioxidant Function Test

##### 2.3.6.1. Measurement of Total Thiol Groups in the Brain

The Tietze method was used to measure the total thiol groups (23). Briefly, after centrifuging the homogenized whole brain tissue at 15,000 g and 4°C for 10 minutes, 100 µL of the supernatant was transferred to a 96-well microplate. After that, 200 µL of Ellman’s reagent (4 mg DTNB in 10 mL of 10% sodium citrate) was added to each well, and the absorbance was measured at 412 nm using an ELISA plate reader (BioTek, Winooski, VT, USA).

##### 3.3.6.2. Measurement of Ferric Reducing Antioxidant Power

This test is used to assess the brain's ability to reduce Fe_3_+ to Fe_2_+ (24). Freshly prepared buffers were used, including 300 mM acetate buffer (Ph = 3.6), 20 mM FeCl_3_, and 10 mM TPTZ containing 0.031 g of TPTZ in 10 mL of 40 mM HCl. At a temperature of 37 degrees Celsius, the diluted sample was mixed with a freshly prepared reagent in a volume of 10 mL. The absorbance was measured at 593 nm using a spectrophotometer.

#### 3.3.7. Measurement of Malondialdehyde Content

Malondialdehyde (MDA), the primary indicator of LPO in biological membranes, reacts with TBA to produce a red-colored product that can be evaluated either colorimetrically or fluorometrically. In this study, MDA content was measured according to the method described by Buege and Aust (25).

### 3.4. Statistical Analysis

Data is presented as Mean ± SD for the report of four training trials per day. Data from escape latency, swimming speed, and travel distance tests were analyzed using a 4 × 4 two-way analysis of variance (ANOVA), with the training day and diamorphine doses considered as within-group and between-group factors, respectively. The data from time spent in the target quadrant in the probe test and parameters of mitochondria, including ROS, MMP, swelling, ADP/ATP ratio, and LPO, as well as antioxidant tests, were analyzed using one-way ANOVA. Tukey’s post-hoc analysis was conducted for multiple comparisons. A significance level of P < 0.05 was considered significant.

## 4. Results

### 4.1. Function of Spatial Learning and Memory in MWM

**Figure 1.**
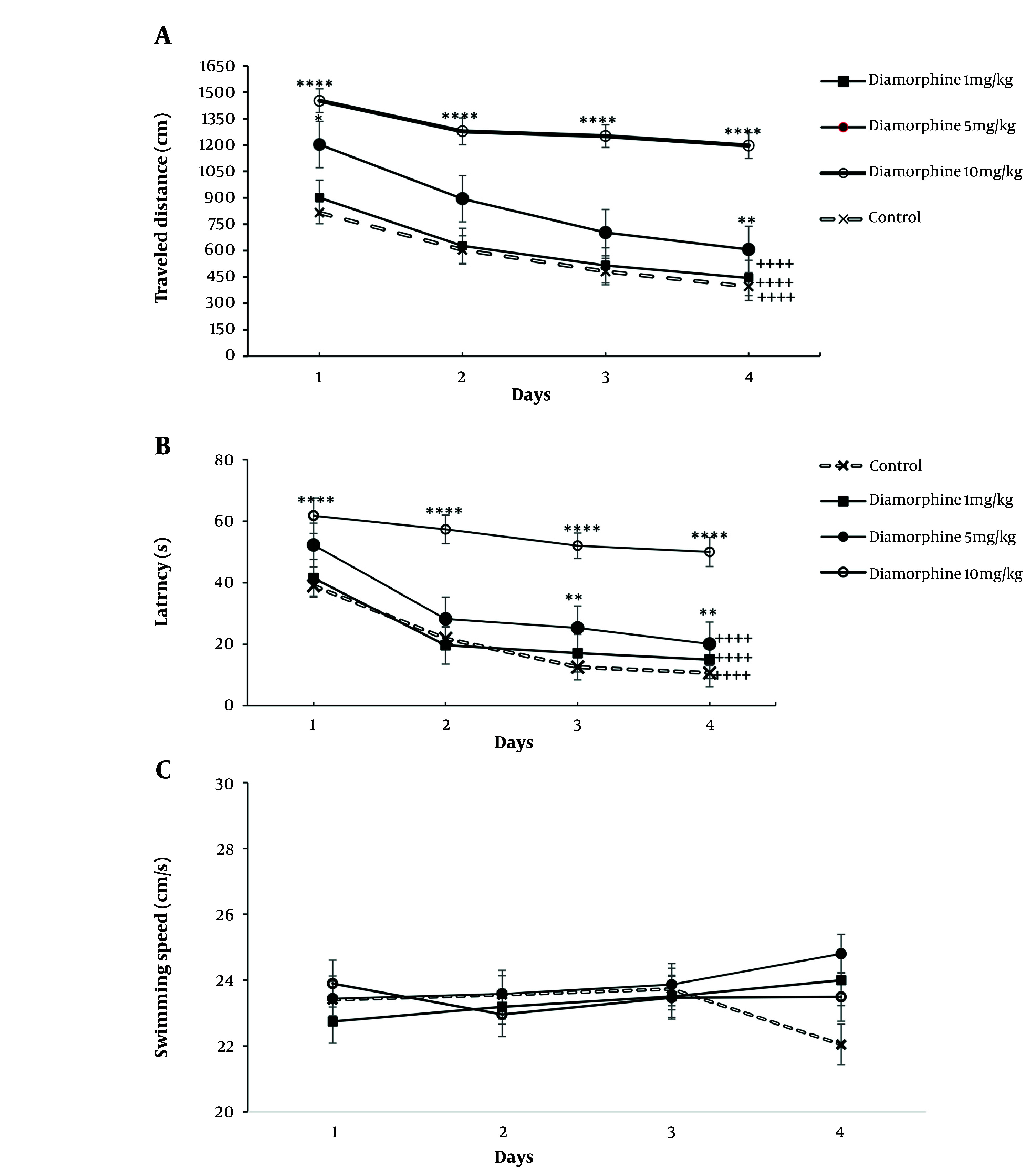
Alterations in the Morris Water Maze test (MWM) results over four training days in groups receiving doses of 1, 5, and 10 mg/kg of diamorphine: A, distance traveled; B, latency; and C, swimming speed (** P < 0.01, and **** P < 0.0001 compared to the control group on the same day; ++++ P < 0.0001 compared to day 1 in the same group).

The results of the statistical analysis using a two-way ANOVA test showed that the dose of diamorphine and the number of days of training had a significant effect on the time taken and distance traveled to reach the platform. Further analysis through multiple comparisons revealed a significant decrease in both distance traveled and time taken over a span of 4 days in both the control group and the groups that received diamorphine, except for the 10 mg/kg group. Additionally, the 10 mg/kg dose diamorphine group consistently traveled a longer distance and took more time to reach the platform compared to the control group on all training days (Figure 1A). Latency (dose: F(3, 28) = 666.5, P < 0.0001, η^2^_p_ = 0.98), latency (training days: F(3, 84) = 90.39, P < 0.0001, η^2^_p_ = 0.72) (Figure 1B). Distance (dose: F(3, 28) = 137.7, P < 0.0001, η^2^_p_ = 0.81), distance (training days: F(3, 84) = 90.75, P < 0.0001, η^2^_p_ = 0.66) (Figure 1B). Furthermore, the interaction effect of diamorphine dose and training days significantly impacted arrival time and distance traveled to the platform. Latency (F(9, 84) = 4.56, P < 0.0001, η^2^_p_ = 0.31), distance (F(9, 84) = 4.8, P < 0.0001, η^2^_p_ = 0.33) (Figure 1B). 

**Figure 2.**
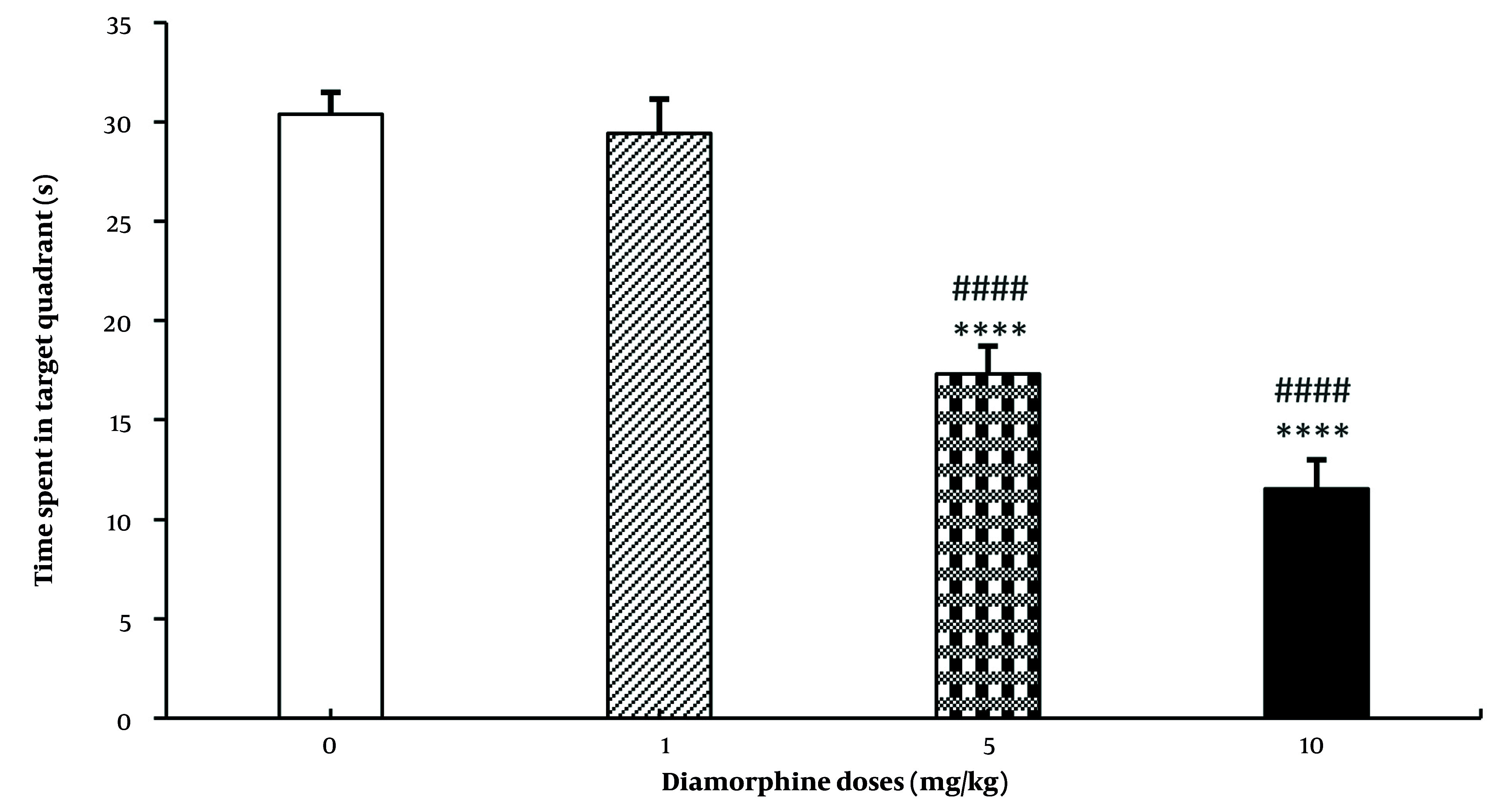
The effect of various doses of Diamorphine on the time spent in the target quadrant in probe test of the Morris Water Maze test (MWM) (**** P < 0.0001 compared to the control group; #### P < 0.0001 compared to the Diamorphine 1 mg/kg group).

For the speed of reaching the platform, neither diamorphine doses nor training days had a significant effect on this variable. Additionally, the interaction effect of diamorphine dose and training days was not statistically significant. Velocity (dose: F(3, 28) = 0.38, P = 0.65, η^2^_p_ = 0.273), Velocity (training days: F(3, 84) = 0.37, P = 0.69, η^2^_p_ = 0.49), Velocity (interaction: F(9, 84) = 0.71, P = 0.58, η^2^_p_ = 0.138) (Figure 1C). The dose of diamorphine significantly affected the time spent in the target area during the probe test on the day of the test in the MWM test (F(3, 31) = 199.24, P < 0.0001, η^2^_p_ = 0.81). Additionally, in terms of time spent in the target area, the doses of 5 and 10 mg of diamorphine spent less time compared to the control group and the dose of 1 mg/kg. The dose of 10 mg/kg also spent less time compared to 5 mg/kg (Figure 2). 

### 4.2. Results of Brain Mitochondria Functional Tests

#### 4.2.1. Reactive Oxygen Species Level of Brain Mitochondria

**Figure 3.**
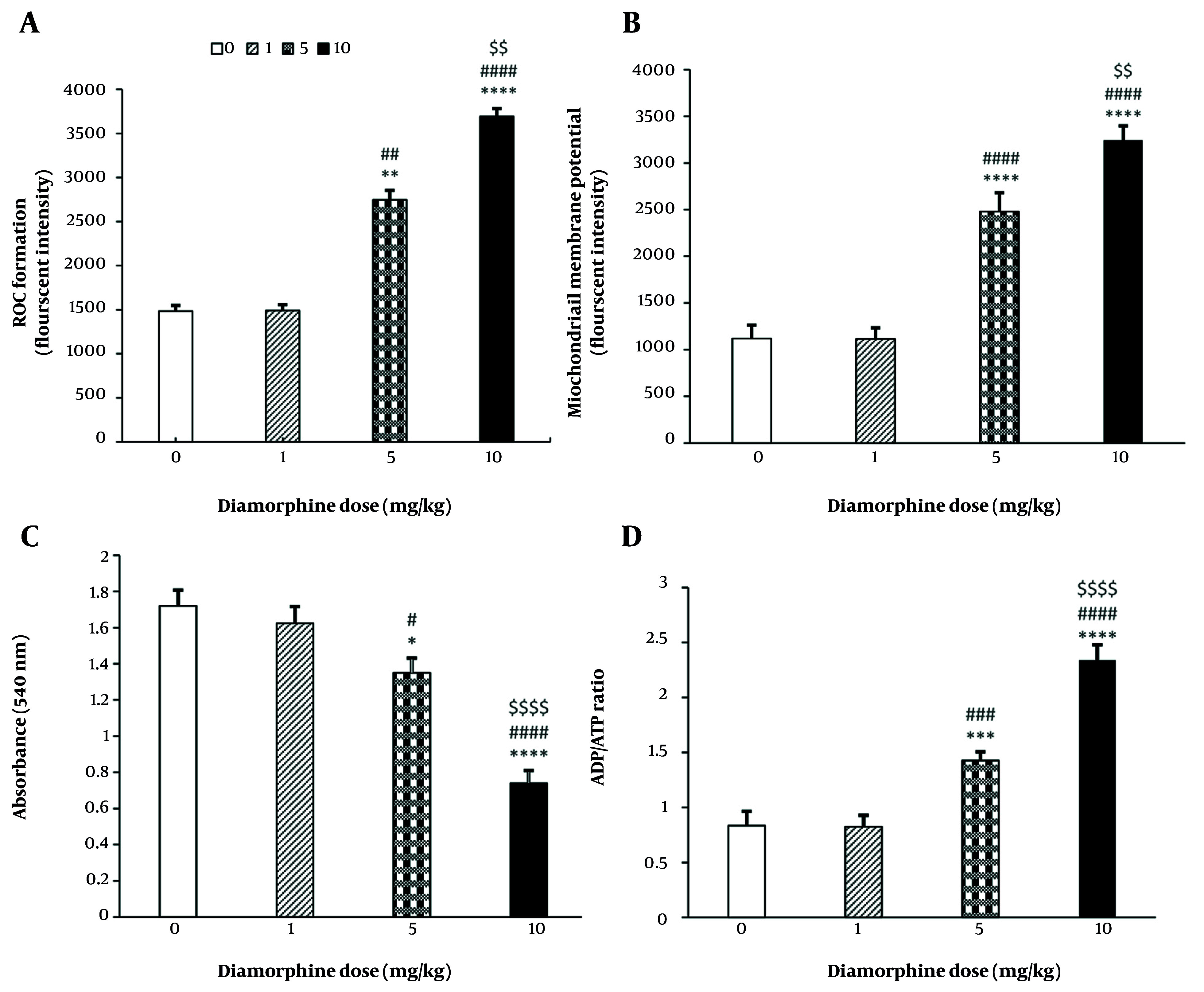
The effect of different doses of Diamorphine on mitochondrial and cellular parameters: A, reactive oxygen species (ROS) formation; B, mitochondrial membrane potential (MMP); C, mitochondrial swelling; D, brain ADP/ATP ratio (* P < 0.05, *** P < 0.001, **** P < 0.0001 compared with the control group; ## P < 0.01, ### P < 0.001, and #### P < 0.0001 compared with the diamorphine 1 mg/kg group; $$ P < 0.01 and $$$$ P < 0.0001 compared with the diamorphine 5 mg/kg group).

The results have shown a direct relationship between the dose of diamorphine and the level of mitochondrial oxidative stress in the brain. The level of oxidative stress significantly increases with the dose (F(3, 19) = 848555, P < 0.0001, η^2^_p_ = 0.93), as represented in Figure 3A. Multiple comparisons indicate significantly higher brain mitochondrial oxidative stress in dose groups 5 mg/kg (P < 0.01) and 10 mg/kg (P < 0.0001) compared to the control group, as well as in the 10 mg/kg group compared to the 1 mg/kg and 5 mg/kg groups (P < 0.0001, P < 0.01). However, there were no significant differences between the 1 mg/kg group and the control group in brain mitochondrial ROS levels (P = 0.191).

#### 4.2.2. Measuring the Mitochondrial Membrane Potential

The results indicate that diamorphine significantly decreases MMP, with higher doses likely causing a greater decrease (F(3, 19) = 180686, P < 0.0001, η^2^_p_ = 0.93). Multiple comparisons have shown that the level of MMP is significantly lower in dose groups of 5 mg/kg (P < 0.0001) and 10 mg/kg (P < 0.0001) compared to the control group. Additionally, the 10 mg/kg group showed significantly lower levels compared to the groups that received 1 mg/kg and 5 mg/kg of diamorphine (P < 0.0001, P < 0.01). This significant difference is reflected in the intensity of fluorescence absorption in the graph. Conversely, diamorphine at 1 mg/kg did not have a significant effect on MMP compared to the control group (P = 0.191) (Figure 3B). 

#### 4.2.3. Measuring the Amount of Mitochondrial Swelling

The results indicated that the impact of diamorphine dosage on brain mitochondrial swelling was significant (F(3, 19) = 2799, P < 0.0001, η^2^_p_ = 0.94). Multiple comparisons revealed that the level of swelling was notably higher in the groups receiving 5 mg/kg (P < 0.05) and 10 mg/kg (P < 0.0001) compared to the control group, and in the 10 mg/kg group compared to the 1 and 5 mg/kg groups (P < 0.0001). Conversely, diamorphine at 1 mg/kg did not have a significant effect on brain mitochondrial swelling compared to the control group (P = 0.188) (Figure 3C). 

#### 4.2.4. Brain ADP/ATP Ratio

The results indicated that the dose of diamorphine has a significant effect on the ADP/ATP level (F(3, 19) = 64.1, P < 0.0001, η^2^_p_ = 0.98). Multiple comparisons revealed that the ADP/ATP levels were significantly higher in the 5 mg/kg dose group (P < 0.001) and 10 mg/kg dose group (P < 0.0001) compared to the control group. Additionally, the 10 mg/kg dose group had significantly higher ADP/ATP levels compared to the 1 and 5 mg/kg groups (P < 0.0001). Conversely, the 1 mg/kg dose of diamorphine did not have a significant effect on ADP/ATP levels compared to the control group (P = 0.182) (Figure 3D). 

### 4.3. The Results of Brain Antioxidant Tests

#### 4.3.1. Total Thiol Groups Measurement

**Figure 4.**
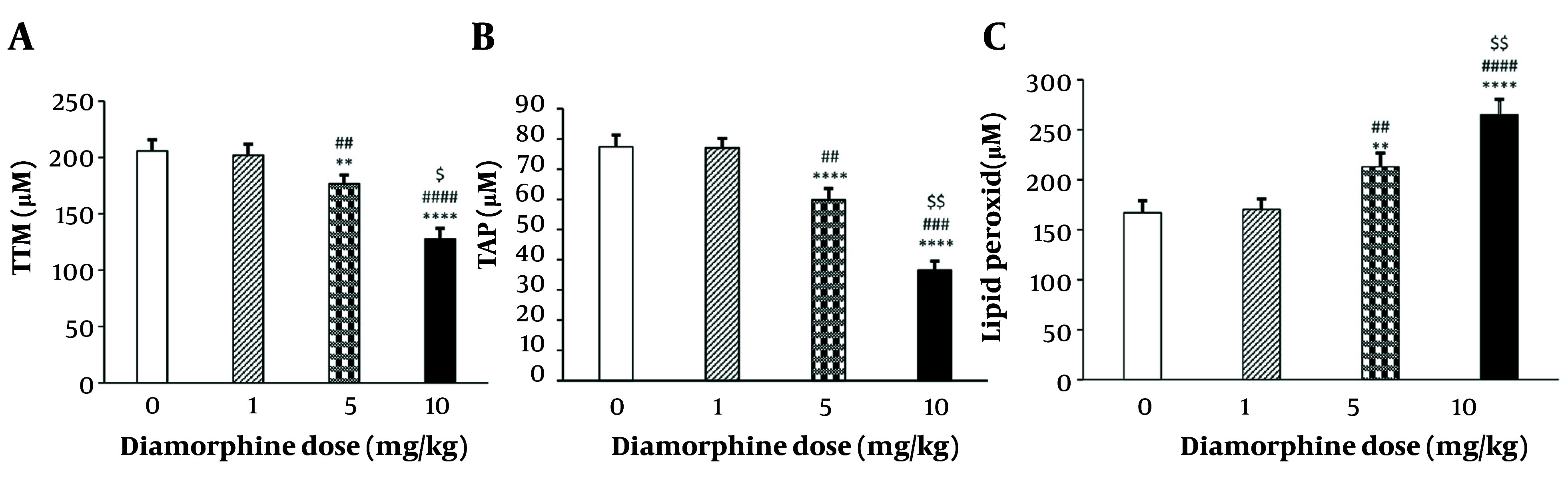
Plot of the effects of various doses of diamorphine on antioxidant tests in the rat brain: A, total thiol groups measurement (TTM); B, ferric reducing antioxidant power (FRAP); C, lipid peroxidation (LPO) (** P < 0.001 and **** P < 0.0001 compared with the control group; ## P < 0.01, ### P < 0.001, and #### P < 0.0001 compared with the diamorphine 1 mg/kg group; $ P < 0.05 and $$ P < 0.01 compared with the diamorphine 5 mg/kg group).

The results showed significant differences in the reduction of the total thiol groups in brain tissue based on the dose of diamorphine administered (F(3, 19) = 82.11, P < 0.0001, η^2^_p_ = 0.89). Multiple comparisons revealed that the levels of TTM significantly decreased in the 5 mg/kg (P < 0.001) and 10 mg/kg (P < 0.0001) dose groups compared to the control group. Additionally, the 10 mg/kg group showed a significant decrease compared to the 1 mg/kg and 5 mg/kg groups (P < 0.0001, P < 0.05). However, the administration of diamorphine at 1 mg/kg did not have a significant effect on TTM compared to the control group (P = 0.287) (Figure 4A). 

#### 4.3.2. Ferric Reducing Antioxidant Power

The results indicated a significant difference in reducing the antioxidant power of ferric in brain tissue between diamorphine doses (F(3 ,19) = 333.13, P < 0.0001, η^2^_p_ = 0.91). Multiple comparisons revealed that this reduction was significantly greater in the 5 mg/kg (P < 0.01) and 10 mg/kg (P < 0.0001) dose groups compared to the control group, and in the 10 mg/kg group compared to the 1 mg/kg and 5 mg/kg groups (P < 0.0001, P < 0.01). However, diamorphine at 1 mg/kg did not have a significant effect on reducing the antioxidant power of ferric in brain tissue compared to the control group (P = 0.149) (Figure 4B). 

### 4.4. Lipid Peroxidation

The results indicated that diamorphine increased the amount of LPO in brain tissue in a dose-dependent manner (F(3, 19) = 1304.2, P < 0.0001, η^2^_p_ = 0.82). Multiple comparisons showed significantly higher levels of LPO in the 5 mg/kg (P < 0.01) and 10 mg/kg (P < 0.0001) dose groups compared to the control group, and in the 10 mg/kg group compared to the 1 mg/kg and 5 mg/kg groups (P < 0.0001, P < 0.01). However, diamorphine at 1 mg/kg did not have a significant effect on LPO compared to the control group (P = 0.221) (Figure 4C). 

## 5. Discussion

The major finding of this study showed that diamorphine significantly disrupts the process of spatial learning and memory. In the Morris Water Maze test, diamorphine increased latency, decreased distance traveled day by day, and reduced time spent in the target quadrant. These results are consistent with a recent study that showed reduced performance of rats exposed to diamorphine in utero during the MWM test (26). Another study showed no difference in the rats exposed to diamorphine vapor in spatial memory and learning MWM test (27), which could be due to the lower bioavailability of diamorphine in the burning and vaporization method (28).

One explanation for these effects is that diamorphine causes mitochondrial dysfunction, as evidenced by increased ROS levels, mitochondrial swelling, altered ADP/ATP ratio, and decreased MMP. Elevated ROS levels can lead to oxidative stress, disrupting the balance between free radical production and the body's antioxidant defenses. This imbalance can damage lipids, proteins, and DNA (29). This study demonstrated spatial memory and learning impairment resulting from 10-day injections of diamorphine twice a day, a close opioid derivative to morphine (30). One possible reason for this impairment may be the negative impact of diamorphine on neuronal mitochondria in brain regions associated with spatial memory, including the hippocampus, prefrontal cortex, posterior parietal cortex, and other areas of the cerebral cortex (31).

The first mechanism that can be proposed for the cognitive problems of diamorphine may be caused by the dysfunction of mitochondria, the results of which are shown below. The results of mitochondrial function tests showed that diamorphine causes a large number of mitochondrial dysfunctions, including increased ROS levels causing oxidative stress that led to LPO, protein damage, reduced antioxidant capacity, decreased MMP, altered ADP/ATP ratio, and mitochondrial swelling. These findings are consistent with other studies on opioids (32).

In this study, it was demonstrated that diamorphine doses of 5 and 10 mg increased the amount of ROS produced by mitochondria, which aligns with the literature. Diamorphine addiction, through activation of μ-opioid receptors, may result in the generation of ROS similar to morphine. This process can trigger downstream effects such as activation of mitochondrial ROS pathways, ultimately leading to the initiation of apoptosis and the caspase-3 cascade (6, 33, 34).

Although our findings indicate increased ROS and mitochondrial dysfunction, key antioxidant enzymes such as SOD, GPx, and catalase were not assessed. This limits our understanding of oxidative stress mechanisms (35, 36). Moreover, although mitochondrial impairment suggests apoptosis or necrosis, no direct tests such as caspase-3 assay, TUNEL, or histology were performed (37, 38). These are important areas for future studies.

Additionally, diamorphine can be metabolized into free radicals (39). Repeated doses of morphine have been shown to cause an increase in dopamine turnover and xanthine oxidation in the striatum. This increase in dopamine oxidative metabolism contributes to an increase in ROS formation (9, 40).

A study on chronic intravenous diamorphine users showed that diamorphine can impair the redox status of erythrocytes (41). Another study demonstrated that diamorphine can induce DNA damage in C57BL/6J mice in the prefrontal cortex and nucleus accumbens by increasing ROS levels (42). An increase in ROS formation was reported in mice in a study that administered intraperitoneal injections of diamorphine for 40 days (3). Another study reported a significant increase in all oxidative damage indices, such as 8-hydroxy-2′-deoxyguanosine (8-OHdG), protein carbonyl groups, and MDA contents in the brains of diamorphine-treated mice (43). A study on human platelets found a strong association between diamorphine addiction and significant levels of oxidative damage (44). Another study also reported an increase in ROS formation in the hepatic mitochondria of Wistar rats and oxidative damage to the liver caused by diamorphine-based substances (45).

The second mechanism could be that brain antioxidant capacity is impaired by diamorphine. Studies have shown that opioids can decrease the brain's GSH content, leading to a reduction in the enzymatic activities of superoxide dismutase, glutathione peroxidase, and glutathione reductase in the hippocampus. This could be a contributing factor to memory impairment (46).

The results of the present study showed a significant decrease in MMP by diamorphine, especially with higher doses. Previous studies have shown that diamorphine causes a decrease in MMP and induces the mitochondrial pathway of apoptosis in cortical neurons in an in vitro study (47). Another study on PC1_2 _cells reported a significant decrease in MMP after treatment with diamorphine (48). The present study is the first to investigate the effects of diamorphine on MMP in vivo and shows its decreasing effect on MMP, which is in agreement with previous in vitro studies that found similar results.

The present study found significant mitochondrial swelling in rats treated with diamorphine. Another study also reported mitochondrial swelling in rats with prolonged diamorphine addiction (49). Additionally, there is a report of mitochondrial swelling in the brain autopsies of human patients with diamorphine addiction (50). This finding has also been reported to be induced by other opioids such as tramadol in rat liver cells, and fentanyl and remifentanil in rat brains (51, 52). One possible reason for the decrease in MMP and mitochondrial swelling could be the disruption of the electron transfer chain and the opening of the mitochondrial permeability transition (MPT) pores, which have been examined in previous studies with other opioids like tramadol (50).

Our study showed that diamorphine causes an increase in the ADP/ATP ratio, which is a result of lower ATP production than its consumption and could inhibit further ATP consumption (53). The present study also showed that diamorphine decreases brain antioxidant capacity in the tests TTM, FRAP, and LPO. This defect leads to damage in the brain, which is prone to oxidative damage (54). A decrease in total thiol and FRAP was also evident in the groups of rats that received 5 mg/kg or 10 mg/kg of diamorphine, indicating a reduction in brain antioxidant capacity. Studies have shown that overall antioxidant capacity has decreased in a rat model of diamorphine addiction, observed in the activity of enzymatic and non-enzymatic antioxidants such as superoxide dismutase, catalase, and the concentrations of vitamins A, C, and E (55). Our study is the first to examine TTM and FRAP in relation to diamorphine.

The findings of our study showed that MDA significantly increased as an indicator of LPO in the groups of rats that received 5 mg/kg or 10 mg/kg of diamorphine. This could exacerbate oxidative damage to neurons whose antioxidant capacity has already decreased. The MDA accumulation itself damages the mitochondria as an oxidative agent (56). A study showed an increase in LPO among chronic abusers of diamorphine (57).

### 5.1. Conclusions

The findings of this study showed that diamorphine can cause impairment of spatial learning and memory through the impairment of mitochondrial function, including increased ROS production, which causes oxidative stress, leading to LPO, protein damage, and reduced antioxidant capacity. Additionally, mitochondrial dysfunction, characterized by MMP disorder and mitochondrial swelling, along with disruption of the ATP:ADP ratio, results in neuronal damage and changes in memory and learning. This impairment was more prominent with higher doses of diamorphine, especially at the dose of 10 mg/kg. Further studies are needed to elucidate the effects of diamorphine on superoxide dismutase and cytochrome C, as well as to evaluate the effects of antioxidants in preventing these damages.

## Data Availability

The dataset presented in the study is available on request from the corresponding author during submission or after publication.
